# The gut microbiome and irritable bowel syndrome

**DOI:** 10.12688/f1000research.14592.1

**Published:** 2018-07-09

**Authors:** Stacy Menees, William Chey

**Affiliations:** 1 Division of Gastroenterology, Michigan Medicine, 1500 East Medical Center Drive, Ann Arbor, MI 48109-5362, USA; 2 VA Ann Arbor Healthcare System, 2215 Fuller Road, Ann Arbor, MI 48105, USA

**Keywords:** microbiome, metabolome, abdominal pain, diarrhea, bloating

## Abstract

Irritable bowel syndrome (IBS) is one of the most common functional gastrointestinal disorders encountered in clinical practice. It is a heterogeneous disorder with a multifactorial pathogenesis. Recent studies have demonstrated that an imbalance in gut bacterial communities, or “dysbiosis”, may be a contributor to the pathophysiology of IBS. There is evidence to suggest that gut dysbiosis may lead to activation of the gut immune system with downstream effects on a variety of other factors of potential relevance to the pathophysiology of IBS. This review will highlight the data addressing the emerging role of the gut microbiome in the pathogenesis of IBS and review the evidence for current and future microbiome based treatments

## Introduction

**Figure 1.**
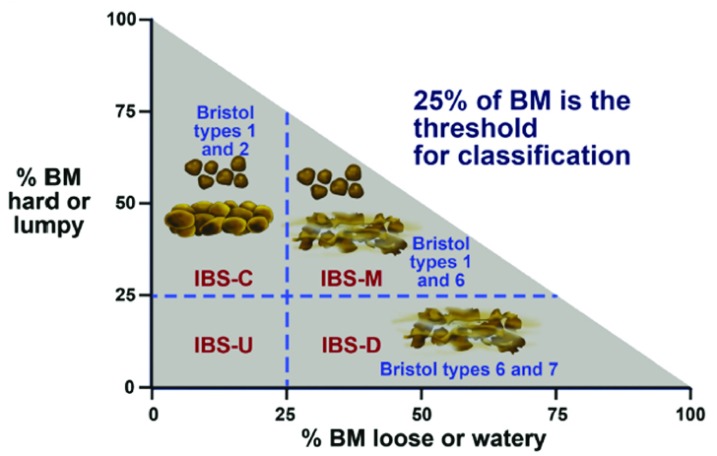
Rome IV criteria for irritable bowel syndrome. ▪   Presence of abdominal pain at least 1 day per week in the last 6 months associated with defecation or a change in bowel habit. ▪   Abdominal pain should meet at least two of three criteria: 1.   Related to defecation 2.   Associated with a change in frequency of stool 3.   Associated with a change in form (appearance) of stool Irritable bowel syndrome (IBS) subgroups are based on stool consistency as measured by the Bristol Stool Form Scale: Those with hard or lumpy stool more than 25% of the time have IBS with constipation, or IBS-C. Those with loose or watery stool more than 25% of the time have IBS with diarrhea, or IBS-D. Those with a mixture of hard or lumpy stools and loose or watery stools have IBS with a mixed bowel pattern, or IBS-M. BM, bowel movement. From 1.

Irritable bowel syndrome (IBS) is a functional bowel disorder defined by the presence of recurrent episodes of abdominal pain associated with altered bowel habits. The recently updated Rome IV criteria are widely regarded as the gold standard of symptom-based criteria ^1^ ( Figure 1). IBS is one of the most commonly encountered gastrointestinal (GI) problems in clinical practice; the prevalence is 12% in the general population ^2^. As measured by validated survey instruments such as the Short Form-36 (SF-36), IBS has a negative impact on an affected patient’s quality of life ^3^. Indeed, IBS reduces health-related quality of life (HRQOL) measured by SF-36 to a greater degree than either diabetes mellitus or end-stage renal disease. Additionally, patients with IBS account for increased resource utilization and decreased productivity compared with healthy persons. Annually, IBS costs the US health system in excess of $30 billion ^4^.

IBS is a disorder of heterogeneous pathogenesis and clinical phenotype. Classically, the pathophysiology for IBS was thought to stem from abnormal brain–gut interactions, visceral hypersensitivity, altered gut motility, and psychological stressors. However, recent evidence implicates a range of other factors as potentially important to IBS, including alterations in gut immune activation, intestinal permeability, and gut microbiome. This update will briefly review the data addressing the emerging role of the gut microbiome in the pathogenesis of IBS and how this rapidly expanding database might provide the substrate for novel diagnostic and treatment strategies.

## The gut microbiome

The microorganisms that inhabit the human GI trace number up to 100 trillion and most inhabit the distal small bowel and colon. Although much attention has been focused on bacteria, it is important to remember that viruses, fungi, archaea, and eukaryotes also contribute to the communities that inhabit the microenvironment of the GI tract ^5– 7^. Studies have demonstrated more than 2,000 different species of bacteria from 12 phyla, and 93.5% of the species are from four dominant phyla: Firmicutes, Bacteroidetes, Proteobacteria, and Actinobacteria ^8, 9^. Recent research suggests that environmental factors such as diet, drugs, and lifestyle exert a greater influence on the gut microbiome than genetics. Furthermore, the gut microbiome may possess a greater ability to predict clinical phenotype and metabolic variables than genetics ^10^.

Bacteria are critical for normal gut development and health. For example, germ-free animals demonstrate delayed gastric emptying and intestinal transit, reduced migrating motor complex cycling and propagation, and reduced *GABA* and *VAP-33* gene expression for the enteric nervous system when compared with animals raised in a normal laboratory environment ^11^. Bacteria also contribute to the health of the host by providing essential amino acids, vitamins, and short-chain fatty acids as well as promoting normal development and function of the intestinal immune system.

## Altered microbiome/and irritable bowel syndrome

The prevailing hypothesis is that an imbalance in gut bacterial communities, or “dysbiosis”, leads to activation of the gut immune system and potential low-grade inflammation ^12^. A key argument supporting this hypothesis is the dramatically increased risk of developing IBS after acute gastroenteritis ^13^. The increased risk of developing so-called “post-infection” IBS is agnostic to the type of infection (bacteria, viruses, or parasites) ^14^. This argues that a range of infectious triggers can activate the immune system in an individual with the right combination of susceptibility factors. Additionally, multiple studies have demonstrated differences in the composition of the gut microbiome within a subset of patients with IBS compared with healthy controls ^15, 16^. Recent work using 16S ribosomal RNA-targeted pyrosequencing and machine learning found a gut microbiome signature which identified with severe IBS ^17^. Furthermore, the diversity and stability of gut microbiota may be reduced in patients with IBS ^18, 19^. Recent data suggest that the community of fungi or “mycobiome” is also altered in patients with IBS and may be associated with the development of visceral hypersensitivity ^20^.

## Small intestinal bacterial overgrowth and irritable bowel syndrome

**Figure 2.**
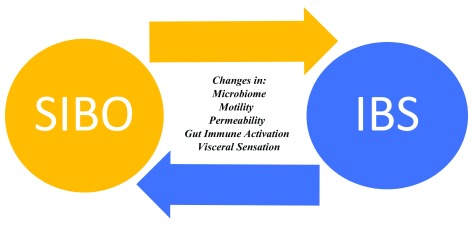
Small intestinal bacterial overgrowth: the chicken or the egg? IBS, irritable bowel syndrome; SIBO, small intestinal bacterial overgrowth.

Small intestinal bacterial overgrowth (SIBO) can induce a wide range of clinical manifestations ranging from mild, vague GI symptoms to frank malabsorption through effects on GI motility ^21, 22^, visceral sensation ^23^, immune activation, carbohydrate digestion and absorption ^24^, bile acid metabolism ^25, 26^, and intestinal epithelial permeability ^27, 28^. Because many of these abnormalities have also been implicated in the pathogenesis of IBS, the possibility of an association between SIBO and IBS is quite intuitive. The lack of an adequately validated gold standard for the diagnosis of SIBO makes it difficult to provide a precise prevalence estimate in patients with IBS. Studies have found that patients with IBS have higher bacterial counts in the proximal small intestine by quantitative culture than healthy controls. We also know that patients with IBS are more likely than healthy volunteers to have an abnormal breath test for SIBO ^29^. What remains unknown is whether SIBO is a cause or a consequence of IBS—or both. In other words, it is biologically plausible to suggest that SIBO can cause IBS symptoms in some but that, in others, alterations in motility, gut immune function, or microbiome predispose to the development of SIBO. If this is true, it is not difficult to imagine how one hand would feed the other, leading to a vicious cycle ( Figure 2). The efficacy of non-absorbable antibiotics in a subset of patients with IBS provides indirect evidence of the relationship between SIBO and IBS. More direct and thus more persuasive evidence of this association is provided by recent studies which report a significantly greater likelihood of clinical response to oral antibiotics in IBS patients with a positive rather than a negative duodenal aspirate for quantitative culture or lactulose breath test ( 16, 17; see “Antibiotics” section below).

## Microbiome-based treatments for irritable bowel syndrome

### Prebiotics

Prebiotics are undigestible oligosaccharides and polysaccharides—fructooligosaccharides or galactooligosaccharides (GOS)—that promote the growth or activity (or both) of bacteria that impart a health benefit for the host. Early work demonstrated that selected prebiotics promoted the growth of potentially beneficial bifidobacteria while inhibiting the growth of potentially harmful Bacteroides, Clostrida, or Coliforms ^30^. A study by Olesen and Gudmand-Hoyer assessed the effect of high-dose inulin (20 g/day) versus placebo for 12 weeks in patients with IBS ^31^. Initial treatment with inulin worsened IBS symptoms in all patients; however, after 12 weeks of treatment, symptoms improved in 58% of the inulin group versus 65% of the placebo group and symptoms worsened in 8% of the inulin group versus 13% of the placebo group, suggesting some level of adaptation in the inulin group. Several other studies using different prebiotics have demonstrated benefit compared with placebo in patients with IBS. Paineau *et al*. performed a double-blind, placebo-controlled trial in 105 IBS subjects with a short-chain inulin-type fructan dosed at 5 g/day over the course of 6 weeks ^32^. Treatment with the prebiotic reduced the intensity of IBS symptoms and improved quality of life as compared with the placebo. Using GOS, Silk *et al*. randomly assigned 44 patients with IBS into three groups: receiving 7 g/day GOS, 3.5 g/day GOS and 3.5 g/day placebo, or 7 g/day placebo for 6 weeks ^33^. The prebiotic significantly improved the composite symptom score, bloating and flatulence, and subject’s global assessment. In those patients receiving GOS, the proportion of bifidobacteria increased in fecal samples. In another study, a novel medical device containing a film-forming agent reticulated protein and a prebiotic mixture of vegetable oligosaccharides and polysaccharides was tested in a multicenter, randomized, placebo-controlled trial ^34^. The researchers found a reduction in abdominal pain ( *p* = 0.017) and flatulence ( *p* = 0.037) with an improvement in quality of life of patients receiving the active treatment ( *p* < 0.0001). Thus, the body of evidence supporting a role for prebiotics as a treatment for IBS is growing. The key will be to understand the dose and duration of prebiotic therapy which encourages the desired effects on the microbiome and improves IBS symptoms without triggering significant symptoms.

### Probiotics

Probiotics are live or attenuated microorganisms that alter gut microbial communities in a way that imparts a health benefit to the host. In the case of IBS, probiotics have been suggested to reduce visceral hypersensitivity or exert anti-inflammatory effects ^35– 37^. Probiotics have been extensively studied in IBS patients with variable effects on gut symptoms. The most recent meta-analysis by Ford *et al*. demonstrated efficacy in IBS patients for improvement of global symptoms, abdominal pain, bloating, and flatulence with a number needed to treat (NNT) of seven ^38^. The relative risk for persistent IBS symptoms for probiotics versus placebo was 0.79 (95% confidence interval [CI] 0.70–0.89). However, this meta-analysis noted that the available evidence could not support recommendations for specific species/strains or combinations of probiotics to treat IBS.

Patients with IBS often have co-morbid psychological distress such as depression or anxiety. Recent studies suggest that IBS and depression share abnormalities in pathophysiology, including dysbiosis, altered intestinal permeability, and gut immune activation ^39^. A number of studies have reported beneficial effects of probiotics on psychological symptoms in healthy individuals ^40^. A recent randomized controlled trial found that a “psychobiotic” containing *Bifidobacterium longum* for 6 weeks improved depression but not anxiety or GI symptoms in patients with IBS to a greater degree than placebo ^41^. Improvements in depression were associated with changes in brain activation pattern by functional magnetic resonance imaging in the “psychobiotic” group.

Further research is required to establish the optimal single- and multi-strain probiotics for IBS. It is almost certain that host characteristics will influence the likelihood that a specific probiotic will benefit a specific patient with IBS. Understanding and leveraging such predictors of response will be key to optimizing the benefits of probiotics for patients with IBS.

Providers and patients should be aware that, depending on the claims made by a manufacturer, probiotics can be regulated in the US as a food, dietary supplement, medical food, or drug. This has implications regarding the purity and likelihood that the product contains viable organisms at the time of purchase. For example, dietary supplements are not required to demonstrate safety or efficacy, and there is no need for US Food and Drug Administration (FDA) approval prior to introduction into the marketplace. On the other hand, medical foods and drugs require a higher level of evidence to achieve regulatory approval by the FDA.

### Antibiotics

The concept of Yin and Yang would seem to apply to the role of antibiotics in IBS. On the one hand, broad-spectrum antibiotics have been shown to negatively impact the gut microbiota by reducing diversity and potentially beneficial bacteria ^42, 43^. Additionally, there is an association between prior use of macrolides ( *p* = 0.036) and tetracycline ( *p* < 0.025) within 12 months and a new diagnosis of IBS ^44^.

On the other hand, there is a robust body of evidence to suggest that non-absorbable antibiotics lead to significant symptom improvement in a subset of patients with IBS. In a meta-analysis of five studies and 1,803 participants, Menees *et al*. demonstrated that rifaximin was more efficacious than placebo for global IBS symptom improvement (odds ratio [OR] = 1.57, 95% CI = 1.22–2.01, therapeutic gain = 9.8%, NNT = 10.2) and bloating (OR = 1.55, 95% CI = 1.23–1.96, therapeutic gain = 9.9%, NNT = 10.1) ^45^. The more recently published Target 3 study reported that 44% of 2,438 patients with IBS-diarrhea (IBS-D) treated with open-label rifaximin (550 mg three times a day for 14 days) experienced a significant improvement in their IBS symptoms. Of those patients who responded to rifaximin, almost 60% developed recurrent IBS symptoms within 18 weeks. In those patients who recurred, retreatment with rifaximin (possible two courses of treatment) led to a significantly greater proportion of responders than placebo ^46, 47^. Overall, the short-term adverse event profile with rifaximin is similar to that of placebo, and stool analyses from the Target 3 study demonstrate short-term depression of diversity and richness ^48^ across a broad range of microbes which was largely reversed at study end.

The randomized trials teach us that an empiric course of rifaximin will lead to improvement in fewer than half of IBS-D patients with an NNT of 10. In addition, most responders will recur within a median of 10 weeks, necessitating repeated courses of rifaximin ^46^. Finally, variable insurance coverage and high acquisition cost create further barriers to the use of rifaximin. Given these issues, a biomarker that could significantly enrich the likelihood of response of IBS-D patients to rifaximin would be welcome ^32^. Recent studies suggest that identifying IBS patients with bacterial contamination of the small intestine, by either aspiration for quantitative culture or lactulose breath testing, may substantially increase the likelihood of response to oral antibiotics ^49– 51^. Although these studies should be viewed as preliminary and hypothesis generating, they provide evidence that at least some of the benefit of oral antibiotics is derived from effects in the small intestine.

### Diet

There has been a surge of interest in dietary interventions to reduce IBS symptoms. Although benefits have been attributed largely to reductions in colonic fermentation or decreased antigen activation of the gut immune system, it is important to consider that diet significantly impacts the composition of the gut microbiome ^52– 54^. For example, reduction in the intake of foods that are high in fermentable oligosaccharides, disaccharides, and monosaccharides and polyols (FODMAP) reduces GI symptoms and improves disease-specific quality of life in patients with IBS ^55– 58^. A recent review of low-FODMAP dietary therapy suggests that at least 50% of patients with IBS report symptomatic benefit ^59^.

The mechanisms by which the low-FODMAP diet improves IBS symptoms are likely multifold; however, there is evidence that alterations in the gut microbiome may play a role. Zhou *et al*. demonstrated that rats fed FODMAPs developed changes in gut microbiota, intestinal permeability, and fecal lipopolysaccharide levels that were associated with the development of visceral hypersensitivity. These abnormalities were reversed by a low-FODMAP diet ^60^. On the other hand, some researchers have expressed concerns about the impact of the low-FODMAP diet on the gut microbiome. Recent studies have demonstrated reductions in potentially beneficial fecal bifidobacteria and butyrate levels in IBS patients on a low-FODMAP diet ^61^. Clearly, further studies assessing the long-term impact of a low-FODMAP diet on the gut microbiome in patients with IBS are needed. In the meantime, it is critical for providers recommending the low-FODMAP diet to recall that elimination is the first of a three-step diet plan. The elimination phase of the diet plan should be viewed as a diagnostic test to identify patients who are sensitive to FODMAPs. Those who respond to a 2- to 6-week trial of FODMAP exclusion should be instructed to reintroduce foods containing individual FODMAPs to determine their sensitivities and allow diversification of their diet in the hopes of improving adherence and minimizing effects on the microbiome. Recent studies suggest that concurrent administration of probiotics can reduce effects on fecal bifidobacteria levels ^38^ and that the use of α-galactosidase supplements may allow some patients with IBS to tolerate GOS ^62^.

### Fecal microbial transplant

The success of re-establishing intestinal homeostasis with fecal microbial transplant (FMT) in recurrent *Clostridium difficile* infection has inspired investigators with an interest in IBS. The first open-label single-center study involved 13 patients with IBS by Rome III criteria who underwent an esophagogastroduodenoscopy (EGD) to have 50–100 mL donor stool infused into the distal duodenum or proximal jejunum ^63^. A total of 70% of participants reported resolution or improvement of symptoms with one adverse event of transiently increased flatus. Holvoet *et al*. performed an open-label single-center FMT study (unknown route) in 12 patients with refractory IBS (Rome III criteria) with intermittent diarrhea and severe bloating ^64^. A total of 75% achieved adequate relief of global IBS symptoms, and 78% of responders continued to report significant relief at 1 year. Fecal microbial analysis demonstrated a tendency toward higher Streptococcus counts at baseline in donors compared with patients ( *p* = 0.011). There was also a trend for higher baseline counts of Streptococcus in successful donors compared with unsuccessful donors and higher microbiota enrichment in responders. In the last open-label single-center study, Mizuno *et al*. enrolled 10 IBS patients (Rome III criteria) who underwent FMT via colonoscopy ^65^. The primary end point was a change in the Bristol Stool Form Scale, and a clinical response was defined as normalization to types 3 or 4. A total of 60% of participants experienced a clinical response, and fecal samples from responders displayed increased microbial diversity. Interestingly, the authors found that donor abundance of *Bifidobacterium* correlated with the therapeutic efficacy of FMT, but a similar increase in participant *Bifidobacterium* did not correlate with FMT success. Most recently, Johnsen *et al*. completed the first double-blind, randomized, placebo-controlled, single-center study in moderate-to-severe IBS-D or IBS-M (Rome III criteria) participants (n = 83) ^66^. The primary end point was symptom relief of more than 75 points assessed by the IBS Severity Scoring System 3 months after FMT. With either fresh or frozen donor feces for active treatment or the participant’s own feces for placebo, FMT was delivered via colonoscopy. A total of 65% of participants receiving active treatment versus 43% receiving the placebo demonstrated response at 3 months ( *p* = 0.049); 12 months after FMT, 56% of participants receiving active treatment versus 36% of 28 receiving placebo had a durable response ( *p* = 0.075). No serious adverse events were attributed to FMT. However, no microbiota assessment analysis was available for this trial. Most recently, preliminary results from 3 additional randomized, controlled trials were presented at Digestive Diseases Week 2018. A single center trial from Belgium in 64 IBS patients with significant bloating reported a statistically significant benefit of nasojejunal administration of donor stool vs. the patient’s own stool for the primary outcome of adequate relief of IBS and bloating symptoms at 12 weeks (49% vs. 29%, p=0.004) ^67^. Unfortunately, 2 additional randomized, controlled trials reported negative results ^68, 69^. A multi-center study from the US which compared oral ingestion of encapsulated stool from healthy donors or placebo in IBS-D patients found no difference in clinical response rate between groups at week 12 (FMT 48% vs. placebo 63%, p=0.32) ^68^. A final study in 52 IBS patients from Denmark reported no statistically significant benefit in IBS symptoms from encapsulated, orally administered donor stool vs. placebo at 12 weeks ^69^. Thus, the efficacy of FMT for IBS remains to be clearly established. Many questions including mechanism of action, proper donor selection, route of administration, durability of response, and short and long term safety require further study before FMT can be considered a mainstream treatment for IBS.

## Conclusions

Recent work has highlighted the role of the gut microbiome in the normal maturation and functioning of the GI tract. There is a growing body of evidence to support the hypothesis that imbalances in microbial communities (dysbiosis) play a role in the pathophysiology of a subgroup of IBS sufferers. An increasing number of interventions for IBS that target the gut microbiome, including prebiotics, probiotics, non-absorbable antibiotics, diet, and FMT, are being evaluated in clinical trials. Not unlike traditional pharmacologic therapies, treatments targeting the microbiome have shown modest but statistically significant benefits for IBS symptoms over placebo. Some have suggested that the results reflect an intrinsic lack of efficacy of the treatments. However, another way of looking at the results is that IBS is a symptom-based disorder of heterogeneous pathophysiology. Thus, IBS likely represents a number of different diseases that happen to present with the same symptoms. That being the case, individual treatments, which target specific pathways or mechanisms, would be expected to improve symptoms in only a subset of IBS sufferers. From now on, it is going to be critical to identify biomarkers that can be added to symptoms when diagnosing and choosing treatment for patients with IBS. This will allow subgrouping of patients on the basis of pathophysiology rather than symptoms alone and enable a greater likelihood of choosing the right therapy for the right patient. The microbiome provides perhaps the most promising target for such a biomarker-based diagnostic and treatment strategy. Recent randomized controlled trials have found that baseline gut microbiome characteristics identified IBS patients who were more likely to respond to the low-FODMAP diet ^70^. Others have reported promising results involving the metabolome or measurement of stool volatile organic compounds which might be leveraged to develop diagnostics which identify IBS patients in whom specific diet treatments might be most beneficial ^71, 72^. A key to deciphering what is abnormal will be to better understand what constitutes a “healthy” microbiome. Most dysbiosis profiles published are based on unsupervised analysis of 16S sequencing, often with poor separation between groups. Finding clinically impactful solutions will require meticulous and multidisciplinary functional analysis of the microbiome using state-of-the-art metagenomic, transcriptomic, proteomic, and metabolomic analyses. This foundational work would be of relevance not only to IBS research but also to a wide range of other areas, including metabolic syndrome, liver diseases, and inflammatory bowel diseases to name a few.

## Abbreviations

CI, confidence interval; FDA, US Food and Drug Administration; FMT, fecal microbial transplant; FODMAP, fermentable oligosaccharides, disaccharides, and monosaccharides and polyols; GI, gastrointestinal; GOS, galactooligosaccharides; IBS, irritable bowel syndrome; IBS-D, irritable bowel syndrome-diarrhea; NNT, number needed to treat; OR, odds ratio; SF-36, Short Form-36; SIBO, small intestinal bacterial overgrowth.
